# Demographic and economic inequality of antenatal care coverage in 4 African countries with a high maternal mortality rate

**DOI:** 10.1186/s13690-024-01288-3

**Published:** 2024-05-06

**Authors:** Winini Belay, Amanuel Belay, Tariku Mengesha, Mizan Habtemichael

**Affiliations:** 1 Department of Reproductive Health and Health Service Management, School of Public Health, Addis Ababa University, P.O. Box 9086, Addis Ababa, Ethiopia; 2 Centre for Innovative Drug Development and Therapeutic Trials for Africa, College of Health Sciences, Addis Ababa University, Addis Ababa, Ethiopia; 3 Department of Epidemiology, St. Peter Specialized Hospital, Addis Ababa, Ethiopia; 4 College of Health Sciences, Addis Ababa University, Addis Ababa, Ethiopia

**Keywords:** Antenatal coverage, Demographic inequality, Economic inequality, African countries

## Abstract

**Background:**

Maternal deaths are concentrated in low and middle-income countries, and Africa accounts for over 50% of the deaths. Women from socioeconomically disadvantaged households have higher morbidity and mortality rates and lower access to maternal health services. Understanding and addressing these inequalities is crucial for achieving the Sustainable Development Goals and improving maternal health outcomes. This study examines the demographic and economic disparities in the utilization of antenatal care (ANC) in four countries with high maternal mortality rates in Africa, namely Nigeria, Chad, Liberia, and Sierra Leone.

**Method:**

The study utilised data from Demographic and Health Surveys (DHS) and Multiple Indicator Cluster Surveys (MICS) from Nigeria, Chad, Liberia, and Sierra Leone. The data was obtained from the Health Equity Assessment Toolkit (HEAT) database. The study examined ANC service utilisation inequality in four dimensions such as economic status, education, place of residence, and subnational region across different subgroups by using four summary measures (Difference (D), Absolute Concentration Index (ACI), Population Attributable Risk (PAR), and Population Attributable Factor (PAF)).

**Result:**

A varying level of inequality in ANC coverage across multiple survey years was observed in Nigeria, Chad, Liberia, and Sierra Leone. Different regions and countries exhibit varying levels of inequality. Disparities were prominent based on educational attainment and place of residence. Higher level of inequality was generally observed among individuals with higher education and those residing in urban areas. Inequality in ANC coverage was also observed by economic status, subnational region, and other factors in Nigeria, Chad, Liberia, and Sierra Leone. ANC coverage is generally higher among the richest quintile subgroup, indicating inequality. Nigeria and Chad show the highest levels of inequality in ANC coverage across multiple measures. Sierra Leone displays some variation with higher coverage among the poorest quintile subgroup.

**Conclusion and recommendation:**

Inequalities in ANC coverage exist across age groups and survey years in Nigeria, Chad, Liberia, and Sierra Leone. Disparities are prominent based on education, residence, and economic status. Efforts should focus on improving access for vulnerable groups, enhancing education and awareness, strengthening healthcare infrastructure, and addressing economic disparities.

**Table:** 

Text box 1. Contributions to the literature
∙ Maternal deaths are a pressing issue in LMICs, with Africa having higher death rates.
∙ Socioeconomic status greatly affects maternal health, leading to higher morbidity and mortality for women from disadvantaged households.
∙ Inadequate access to quality maternal health services contributes to the persistently high maternal mortality rates in Africa.
∙ Efforts should be made to improve access to ANC for vulnerable groups, enhance education and awareness, strengthen healthcare infrastructure, and address economic disparities to improve maternal health outcomes.

## Introduction

The World Health Organization (WHO) defines ANC utilization as a measure of antenatal care coverage, which is calculated as the percentage of women aged 15–49 with a live birth in a given period who received antenatal care of four or more times [1]. The Sustainable Development Goal (SDG) of the World Health Organization(WHO) target between 2021 and 2030 requires achieving and reducing maternal mortality by 11.6% [2]. Many low and middle-income cuntries (LMICs) have significantly improved antenatal care(ANC) coverage [3]. According to WHO, almost all maternal deaths (99%) occur in low and middle-income countries with declining tries (LMICs), among which Africa alone accounts for over 50% of the deaths [4]. Sub-Saharan Africa (SSA) accounted for about 70% of global maternal deaths in 2020 [5]. Poverty and access to health care services are major development problems in Africa [6]. Evidence from SSA countries has revealed that health outcomes and access to key health services are unevenly distributed across different social groups of the population and that women and children from socioeconomically disadvantaged homes have higher morbidity and mortality rates and lower coverage of health [6, 7].

There are various factors influencing the utilization of ANC services, where some of them includes; maternal factors (age, marital status, and education), wealth status, clinical factors (parity and pregnancy complications), and environmental factors such as partner support, media exposure, as well as distance to health facility [8–10]. Women from poorer households generally access far less maternal care [11, 12], while mothers in the highest wealth status are more likely to be assisted by skilled birth attendants(SBA) and have institutional deliveries [4, 13]. A wealth inequality cannot be isolated from the compounding effects of other factors like education and the place of residence [4, 14, 15].

Inequality in maternal health service impedes national progress owing to the direct and indirect losses arising from poor maternal and child health [16]. Socio-economic inequities in maternal and child health are present throughout the world, irrespective of a country’s level of health and wealth [17]. The socioeconomic inequality is a growing research attention in the domain of population health where the economically disadvantaged sections of society are also the ones that suffer the worst health conditions [18, 19]. Intuitively, economic constraints are a strong limiting factor for the accessibility and affordability of healthcare services for mothers from poor households [20]. A disaggregated analysis by socio-economic status (SES) shows huge inequality in the use of ANC service between the poor and rich, with people in the lower end of the socio-economic spectrum suffering from low coverage [21]. Even in the same wealth index category the coverage of first ANC service and at least four visits of ANC were 41.1% and 26.9% respectively [22]. The Population Attributable Risk (PAR) of ANC services highlight the need to have approaches that mainly target people from the poorest SES spectrum to efficiently improve these process indicators without ignoring the need to plan for the whole population approach [19].

Widening ANC service utilization means that many countries are off-track on health and wellbeing-related SDGs and reducing inequalities in all forms. It therefore, remains critical to understand how countries are progressing in increasing ANC coverage and closing the gaps by reducing inequalities between the wealthy and poorer populations to leave no one behind [3]. The social environment and economic circumstances significantly affect a woman’s chances of surviving pregnancy and childbirth [23]. Understanding socioeconomic inequalities in terms of access to effective healthcare services is crucial for designing appropriate evidence-based programs and policies [24]. Thus, the objective of this study was to assess the demographic and economic inequality of Antenatal care coverage in 4 African countries with high maternal mortality rate.

## Methods

### Setting

Nigeria is the most populous country in Africa with approximately 162 million people and expected to continue to grow to 239 million by 2025 and 440 million by 2050 due to population momentum [25]. In Nigeria, over one-third of pregnant women do not attend Antenatal Care (ANC) services during pregnancy [26]. Among women of childbearing age, 34.9% did not use ANC service, and one hundred forty-five Nigerian women die in childbirth every day [27]. Chad is a landlocked country in central sub-Saharan Africa. In 2015, the United Nations Development Program ranked Chad 185 out of 188 countries on the Human Development Index [27]. Chad reports the second highest maternal mortality worldwide, where the healthcare system in Chad suffers from limited resources and infrastructure, leading to disparities in antenatal care coverage among different population groups [28].

**Fig. 1:**
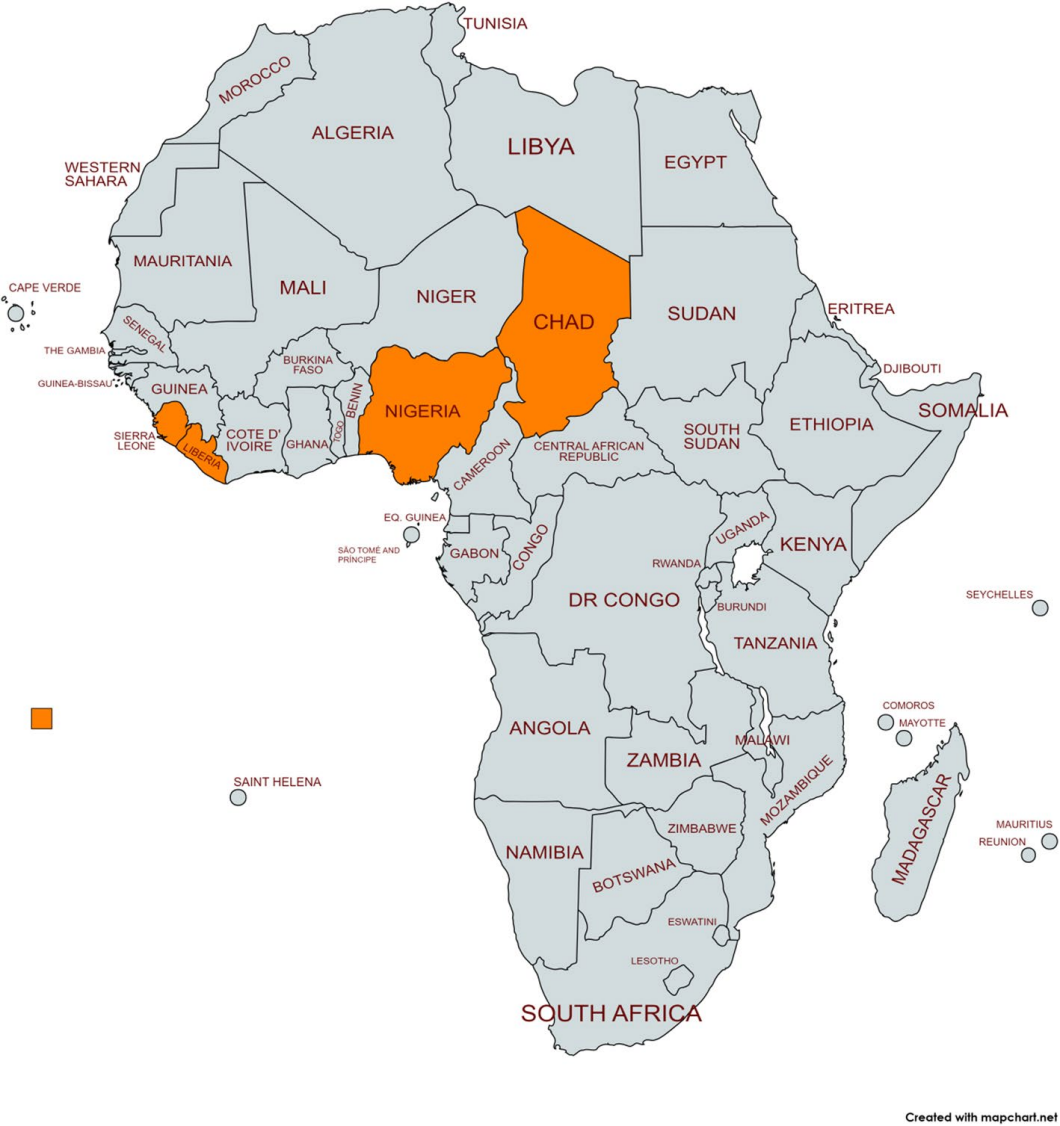
Map of Africa, study countries marked in Orange

Liberia is a country on the west coast of Africa, with highest maternal mortality ratios in the world, where the healthcare system in Liberia is fragile, and access to antenatal care services is limited, especially in remote and rural areas [29]. Sierra Leone is a West African country that has an estimated population of 8.3 million and one of the highest maternal mortality rates globally. The country faces numerous challenges, including inadequate healthcare infrastructure and limited resources, resulting in disparities in antenatal care utilization [30] Fig. 1.

### Data source

The study utilized data from the Demographic and Health Surveys (DHSs) and Multiple Indicator Cluster Surveys (MICSs) accessed from the WHO Health Equity Assessment Toolkit (HEAT) [31], a comprehensive database that provides information on health inequalities and equity within and between countries. The survey tools used by DHS and MICS permit direct comparisons between surveys, and it is assumed that the survey design and implementation quality are sufficiently similar between DHS and MICS, across countries and over time. A methodology of scientific probability sampling is used in DHS surveys. Such a sample is one in which the units are chosen randomly with known and nonzero probabilities, which is necessary for unbiased estimation and error evaluation. Since non-sampling errors (coverage errors, errors from survey implementation and data processing, etc.) are usually more significant and costlier to control, DHS surveys employ a two-stage household-based sample design that is relatively easy to implement and maintain [32]. The sample for the Multiple Indicator Cluster Survey (MICS) was designed to provide national-level estimates of health indicators for urban and rural areas and regions. The sample was selected in two stages: in the first stage, census enumeration areas were chosen with probability proportional to size, and in the second stage, a systematic sample of households was drawn within the selected enumeration areas. The MICS sample was stratified by region, so it was not self-weighting. For reporting national-level results, sample weights are used [33]. It is important to note that MICS and DHS collaborate closely and work through interagency processes to ensure that their survey tools are harmonized and comparable, and their data can be combined in global databases covering a significant portion of developing countries. Therefore, it is very crucial to notice these variations while interpreting the result [34].

The HEAT database includes data on various health indicators, including antenatal care coverage, collected through surveys and other sources. The study includes available data in different years from each of the four study countries. For Nigeria, data from 2003, 2008, 2011, 2013, 2016 and 2018 was used. For Chad, data from 2007, 2013 and 2019 was utilized. Liberia, data from 2007, 2013 and 2019 was used. Lastly, for Sierra Leonne, data from 2008, 2013, and 2019 was utilized. The selection of the study years is solely based on the availability of the data from each survey years in the individual countries. Some countries may conduct surveys or censuses more frequently, while others may have longer intervals between data collection rounds. which creates variation in data points.

### Variables and measurements

The ANC service utilization by women in all of the four countries under study was examined based on four socio demographic and economic dimensions: economic status, education, place of residence, and subnational region. Economic status was determined by assessing the living conditions of households, including ownership of assets such as televisions and bicycles, housing materials, and access to water and sanitation facilities. Principal components analysis (PCA) was used to analyze the economic status, specifically the wealth index, and the relative wealth was divided into five wealth quintiles [35]. Age was categorized as 15–19 years and 20–49 years, Educational status was classified by three categories: (1) no formal education, (2) primary school (3) secondary school. Place of residence was categorized as urban or rural depending on each country’s national classification. Subnational region was classified according to the central government’s administrative system of each country.

### Statistical analysis

Statistical analysis was conducted using HEAT version 3.1 software. Each survey from the four countries of interest was evaluated using four summary measures of inequality. These measures, which combine absolute and relative indicators, were used to assess inequality. The four summary measures employed were: Difference (D), Absolute Concentration Index (ACI), Population Attributable Risk (PAR), and Population Attributable Factor (PAF). The absolute measures (D, ACI, and PAR) provide information about the extent of health disparities between different subgroups and are expressed in the same units as the health indicator being measured. On the other hand, the relative measures and PAF indicate proportional differences in health among subgroups and are dimensionless [36].

### Description of summary measures

D was determined by comparing the ANC coverage rates for at least four visits among different groups based on education, economic status, place of residency, age, and subnational region. The advantaged subgroup, which consisted of individuals with richest wealth quintile, secondary education or higher, age range of 20–49, urban dwellers, or residing in the subnational region with the highest ANC coverage estimate, and had their coverage rate calculated. This rate was then subtracted from the percentage of the disadvantaged subgroup, which included individuals with no formal education, the poorest (lowest wealth quintile), rural dwellers, or residing in the subnational region with the lowest ANC coverage estimate.

The PAR (Population Attributable Risk) was determined by subtracting the estimated ANC (Antenatal Care) coverage for the privileged subgroups (richest wealth quintile, secondary education or higher, age range of 20–49, urban dwellers, or residing in the subnational region with the highest ANC coverage estimate from the overall national average of ANC coverage.

PAF was calculated by dividing the PAR by the national average (μ) and multiplying the fraction by 100, i.e. [PAF = (PAR/μ) × 100].To calculate ACI, the following formula was employed: ACI = ∑jp(2Xj − 1)yj, where (1) yj indicates the estimate of ANC coverage for each subgroup j, (2) p indicates the population share of subgroup j, (3)Xj indicates the relative rank of subgroup j, and relative rank is calculated as: (Xj = ∑jpj − 0.5pj), obtained from a weighted sample of the whole population rank from 0 (most disadvantaged subgroup) to 1 (most advantaged subgroup) [37]. The rationale for using the aforementioned summary measures is that they incorporate both absolute and relative measures, as well as complex-weighted and simple-unweighted measurements. These measures are believed to provide a comprehensive perspective on the data being analyzed. The incorporation of these summary measures is intended to offer a more complete understanding of the data.

## Result

### ANC coverage (at least four visits (%)) by Age

**Fig. 2:**
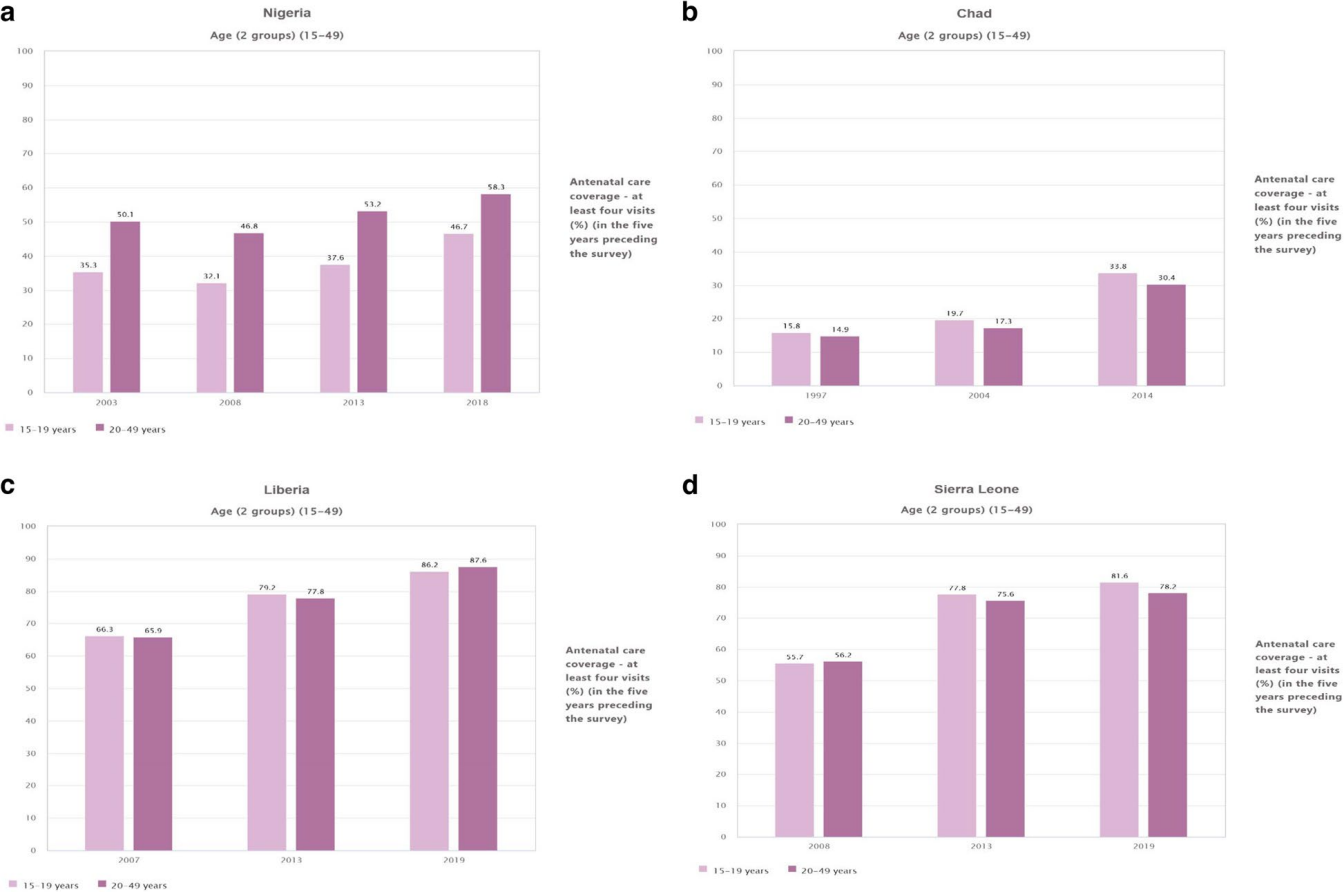
**a** Proportion of ANC coverage at least four visit in Nigeria, by Age (2003,2008,2013 and 2018). **b** Proportion of ANC coverage at least four visit in Chad, by Age 1997,2004 and 2014. **c** Proportion of ANC coverage at least four visit in Liberia, by age (2007, 2013,and 2019). **d** Proportion of ANC coverage at least four visit in Sierra Leone, by age (2008, 2013,and 2019)

There was a notable proportion of ANC coverage for at least four visits among individuals aged 20–49 years in Nigeria in 2003, reaching 50.2% (95% CI, 47.2, 53.2) than those aged 15–19 years. However, in 2008, there was a decline in coverage to 46.8% (95% CI, 45.3, 48.5) (Fig. 2a). In 1997, the proportion of ANC coverage among individuals aged 20–49 years in Chad was 14.9% (95% CI, 13.2, 16.8). However, coverage was pointedly increased in 2004 (17.3%) and 2014 (30.6%). This rise was also observed in the younger age bracket (15–19 year) (Fig. 2b). The ANC coverage among individuals aged 20–49 in Liberia was 66% (95% CI, 62.9, 68.8) in 2007. Subsequently, there was an increase in coverage to 77.9% (95% CI, 75.6, 79.9) in 2013 and further to 87.6% (95% CI, 85.6%, 89.3) in 2019 (Fig. 2c). The ANC coverage in Sierra Leone for individuals aged 20–49 exhibited an upward trend, starting from 56.8% (95% CI, 53.5, 58.9) in 2008 and increasing to 78.2% (95% CI, 76.1, 80.2) in 2019 (Fig. 2d).

### ANC coverage (at least four visits (%)) by educational status

**Fig. 3:**
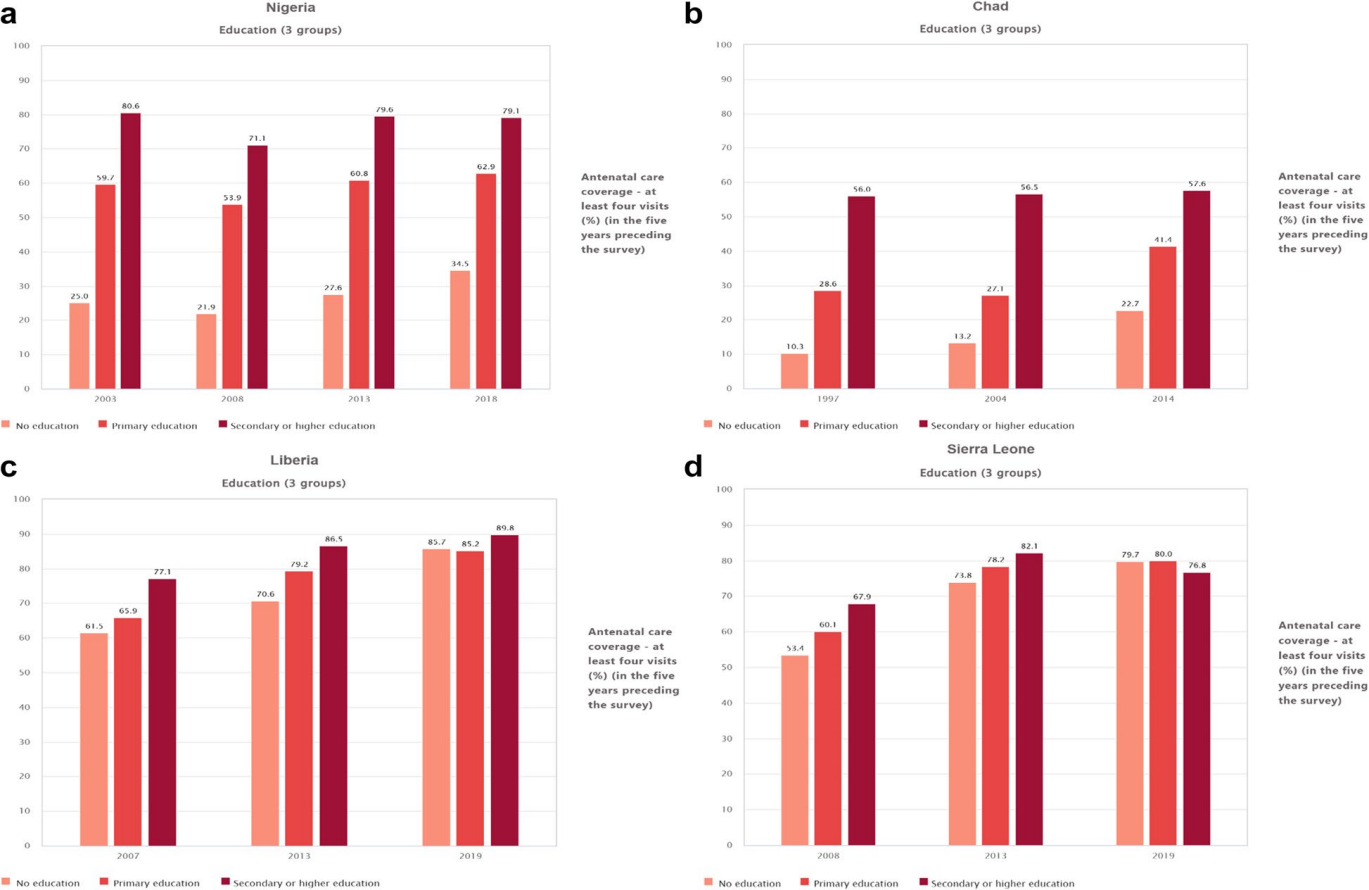
**a **Proportion of ANC coverage at least four visit in Nigeria, by educational status (2003, 2008, 2013 and 2018). **b** Proportion of ANC coverage at least four visit in Chad, by educational status (1997, 2004 and 2014). **c** Proportion of ANC coverage at least four visit in Liberia, by educational status (2007, 2013 and 2019). **d **Proportion of ANC coverage at least four visit in Sierra Leone, by educational status (2008, 2013 and 2019)

The ANC coverage in the survey year 2003 in Nigeria among individuals with no formal education was 25% (95% CI, 22.2, 28.1). However, there was a decrease in coverage among this group in 2008, with a proportion of 21.9% (95% CI, 20.1, 23.9). On the other hand, an ascending trend in coverage was observed in the survey years 2013 (27.6%) and 2018 (34.5%) for individuals with no formal education. A descending trend was observed among other educational status subgroups between 2003 and 2008, followed by ascending trends in 2013 and 2018 (Fig. 3a).

The ANC coverage of at least four visits in Chad exhibited an ascending trend in the survey years 1997, 2004, and 2014 among individuals with no formal education, with proportions of 10.3%, 13.2%, and 22.7% respectively. On the other hand, for individuals with primary education, there was a slight descending trend from the survey year 1997 (28.6%) to the survey year 2004 (27.1%), but an increase in coverage was observed in the survey year 2014 (41.5%). Additionally, an increase in ANC coverage was consistently observed among individuals with secondary educational attainment in all the survey years (Fig. 3b).

The ANC coverage of at least four visits in Liberia has shown an upward trend in all educational subgroups across all survey years. Among individuals with no formal education, the coverage proportion was recorded to be 61.5% (95% CI, 57.4, 65.4) in 2007, 70% (95% CI, 67.2, 73.8) in 2013, and markedly increased to 87.8% (95% CI, 82.9, 88.1) in 2019. It's worth noting that ANC coverage has notably increased throughout all survey years within each educational attainment subgroup (Fig. 3c).

In Sierra Leone, the ANC coverage of at least four visits exhibited an upward trend in all survey years, specifically in subgroups with no formal education and primary education. However, there was a noticeable downward trend in the secondary school educational subgroup from 2013 (82.1% with a 95% CI of 78.5% to 85.1%) to 2019 (76.8% with a 95% CI of 73.1% to 80.1%) (Fig. 3a, b, c, d).

### ANC coverage (at least four visits (%)) by place of residence

**Fig. 4:**
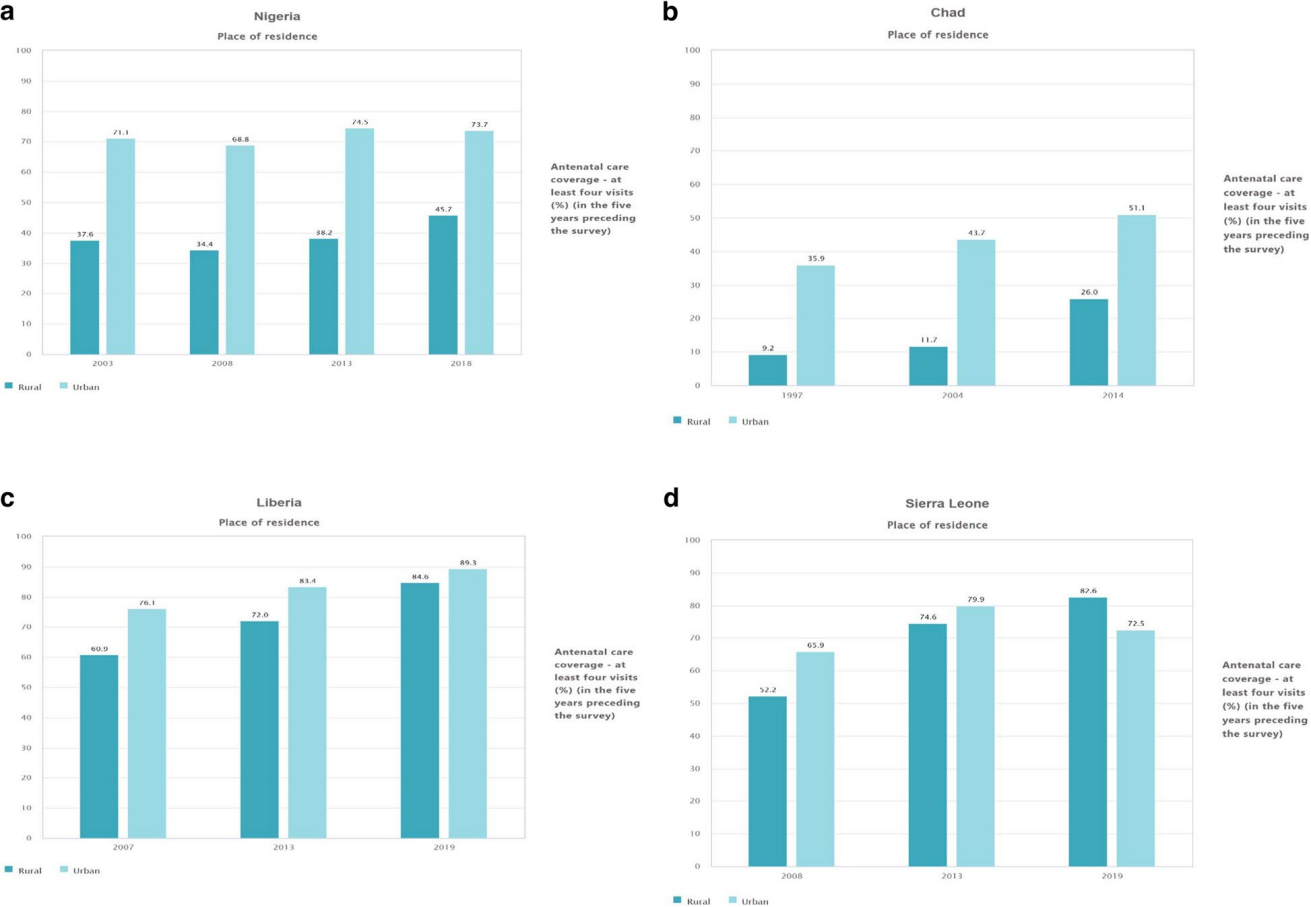
**a** Proportion of ANC coverage at least four visit in Nigeria, by residence (2003, 2008, 2013 and 2018). **b** Proportion of ANC coverage at least four visit in Chad, by residence (1997, 2004 and 2014). **c** Proportion of ANC coverage at least four visit in Liberia, by residence (2007, 2013 and 2019). **d** Proportion of ANC coverage at least four visit in Sierra Leone, by residence (2008, 2013 and 2019)

In 2003 in Nigeria, the ANC coverage in the urban setting is 71.1% while the rural is 37.6%, which shows higher ANC coverage in the urban setting than the rural. While in 2008, the ANC coverage in the urban setting was 68.8% whereas the rural was 34.4%, which indicates higher ANC coverage in the Urban setting than the rural. In the survey year 20,013, the ANC coverage in the urban setting of Nigeria has reached to 74.5% while the rural was 38.2%, this shows high ANC coverage in the urban setting. In the survey year 2018, the ANC coverage in the urban setting of Nigeria was 73.7%, whereas the rural was 45.8%, which shows higher coverage in the urban setting (Fig. 4a). In Chad, the ANC coverage demonstrated an ascending trend in both rural and urban settings across all survey years, depicting higher ANC coverage in the urban setting; 1997(urban = 35.9%, rural = 9.2%), 2004 (urban = 43.7%, rural = 11.7%) and 2014 (urban = 51.1%, rural = 25.9%) (Fig. 4b). Similarly, ANC coverage in Liberia showed an upward trend in all survey years across urban and rural settings; 2007(urban = 76.1%, rural = 60.9%), 2013 (urban = 83.4%, rural = 72.1%) and 2019 (urban = 89.4%, rural = 84.7%) (Fig. 4c). The ANC coverage in Sierra Leone exhibited an upward trend from the survey year 2008 (65.9%) to 2013 (79.9%) in urban settings. However, there was a subsequent descending trend to the survey year 2019 (72.5%), which shows higher ANC coverage in the urban setting than the rural ones (Fig. 4d).

### ANC coverage (at least four visits (%)) by economic status (Nigeria)

ANC coverage of at least four visits in Nigeria showed an ascending trend in all the survey years with the highest coverage identified in the survey year 2018 in which the proportion of quintile 1(poorest) was 30.7%, quintile 2(42.7%), quintile 3(60.6%), quintile 4(73.3%), and quintile 5(richest) was (85.4%). In all study years the ANC coverage was lower for quintile 1 groups and higher among quintile 5 (Table 1).

**Table 1:** Proportion of ANC coverage at least four visits in Nigeria by Economic Status (2003, 2008, 2013 and 2018)

Country	Year	Subgroup	Estimate	SE	95%CI (Lower bound)	95%CI (Upper bound)
Nigeria	2018	Quintile 1 (poorest)	30.67	1.37	28.05	33.42
	2018	Quintile 2	42.65	1.40	39.92	45.42
	2018	Quintile 3	60.63	1.12	58.42	62.80
	2018	Quintile 4	73.24	0.94	71.36	75.04
	2018	Quintile 5 (richest)	85.39	0.86	83.63	86.99
	2013	Quintile 1 (poorest)	17.96	1.34	15.48	20.75
	2013	Quintile 2	34.89	1.45	32.10	37.80
	2013	Quintile 3	57.61	1.40	54.84	60.34
	2013	Quintile 4	72.94	1.27	70.37	75.36
	2013	Quintile 5 (richest)	85.57	0.87	83.77	87.21
	2008	Quintile 1 (poorest)	15.71	1.08	13.70	17.94
	2008	Quintile 2	28.61	1.29	26.14	31.21
	2008	Quintile 3	47.63	1.39	44.92	50.36
	2008	Quintile 4	64.16	1.31	61.55	66.69
	2008	Quintile 5 (richest)	80.65	1.31	77.95	83.10
	2003	Quintile 1 (poorest)	23.56	2.16	19.57	28.08
	2003	Quintile 2	27.71	2.53	23.01	32.96
	2003	Quintile 3	44.57	2.50	39.70	49.54
	2003	Quintile 4	63.86	2.48	58.84	68.60
	2003	Quintile 5 (richest)	88.05	2.57	81.99	92.26

### ANC coverage (at least four visits (%)) by economic status (Chad)

ANC coverage of at least four visits in Chad showed an ascending trend in all the survey years with the highest coverage identified in the survey year 2014 in which the proportion of quintile 1(poorest) was 23.7%, quintile 2(26.2%), quintile 3(26.3%), quintile 4(28.3%), and quintile 5(richest) was (53.4%). The lowest coverage was reported in the survey year 2004 among the quintile 1 (poorest) Economical status subgroup (2.2%, (95% CI, 0.97, 4.9)) (Table 2).

**Table 2:** Proportion of ANC coverage at least four visits in Chad by Economic Status (1997, 2004 and 2014)

Country	Year	Subgroup	Estimate	SE	95%CI (Lower bound)	95%CI (Upper bound)
Chad	2014	Quintile 1 (poorest)	23.74	2.15	19.78	28.21
	2014	Quintile 2	26.21	1.43	23.49	29.12
	2014	Quintile 3	26.25	1.52	23.37	29.36
	2014	Quintile 4	28.29	1.73	25.02	31.8
	2014	Quintile 5 (richest)	53.53	1.53	50.51	56.53
	2004	Quintile 1 (poorest)	2.21	0.91	0.97	4.97
	2004	Quintile 2	11.72	2.93	7.03	18.9
	2004	Quintile 3	11.94	2.1	8.35	16.77
	2004	Quintile 4	20.64	2.49	16.13	26.02
	2004	Quintile 5 (richest)	43.71	2.26	39.29	48.24
	1997	Quintile 1 (poorest)	4.99	0.93	3.44	7.18
	1997	Quintile 2	6.6	0.9	5.04	8.61
	1997	Quintile 3	10.41	1.36	8.01	13.41
	1997	Quintile 4	17.15	1.46	14.46	20.24
	1997	Quintile 5 (richest)	39.85	1.91	36.13	43.68

### ANC coverage at least four visits (%) by economic status in Liberia and Sierra Leone

ANC coverage of at least four visits in Liberia showed an ascending trend in all the survey years with the highest coverage identified in the survey year 2018 in which the proportion of quintile 1(poorest) was 82.5%, quintile 2(87.6%), quintile 3(86.3%), quintile 4(89.2%), and quintile 5(richest) was (91.3%). The lowest coverage was reported in the survey year 2007 among the quintile 1 (poorest) Economical status subgroup (54.9%, (95% CI, 49.5, 60.1)).

The ANC coverage for at least four visits in Sierra Leone exhibited an upward trend in the survey year 2008. The richest subgroup (quintile 5) achieved the highest coverage of 70.1% (95% CI, 64.2, 75.3), while the lowest coverage was 48.8% (95% CI, 44.1, 53.6). However, in the survey years 2013 and 2019, the trend of ANC coverage appeared somewhat irregular (Table 3).

**Table 3:** Proportion of ANC coverage at least four visits in Liberia and Sierra Leone by Economic Status (2007, 2008, 2013 and 2019)

Country	Year	Subgroup	Estimate	SE	95%CI (Lower bound)	95%CI (Upper bound)
Liberia	2019	Quintile 1 (poorest)	82.51	1.57	79.2	85.4
	2019	Quintile 2	87.56	1.37	84.6	90.02
	2019	Quintile 3	86.58	2.01	82.11	90.06
	2019	Quintile 4	89.2	1.79	85.13	92.25
	2019	Quintile 5 (richest)	91.29	1.84	86.91	94.3
	2013	Quintile 1 (poorest)	66.37	1.98	62.37	70.15
	2013	Quintile 2	73.39	1.63	70.06	76.47
	2013	Quintile 3	81.33	1.7	77.74	84.45
	2013	Quintile 4	85.73	1.67	82.13	88.71
	2013	Quintile 5 (richest)	86.83	2.46	81.19	90.96
	2007	Quintile 1 (poorest)	54.85	2.67	49.54	60.06
	2007	Quintile 2	56.67	3.19	50.29	62.83
	2007	Quintile 3	67.91	2.01	63.82	71.74
	2007	Quintile 4	76.27	2.27	71.5	80.47
	2007	Quintile 5 (richest)	78.34	2.2	73.69	82.37
Sierra Leone	2019	Quintile 1 (poorest)	79.43	1.51	76.31	82.23
	2019	Quintile 2	85.22	1.2	82.71	87.42
	2019	Quintile 3	84.03	1.17	81.59	86.2
	2019	Quintile 4	75.35	2.31	70.53	79.61
	2019	Quintile 5 (richest)	67.6	2.78	61.93	72.79
	2013	Quintile 1 (poorest)	73.93	2.08	69.64	77.81
	2013	Quintile 2	72.97	1.86	69.17	76.46
	2013	Quintile 3	76.06	1.64	72.69	79.13
	2013	Quintile 4	78	1.5	74.91	80.81
	2013	Quintile 5 (richest)	80.21	2.47	74.9	84.63
	2008	Quintile 1 (poorest)	48.82	2.41	44.1	53.57
	2008	Quintile 2	52.44	2.14	48.22	56.62
	2008	Quintile 3	55.74	2.61	50.57	60.79
	2008	Quintile 4	56.69	2.19	52.35	60.93
	2008	Quintile 5 (richest)	70.02	2.82	64.2	75.26

### ANC coverage at least four visits (%) by subnational region in Nigeria

As shown in Table 4, the proportion of ANC coverage for at least four visits in Nigeria varied across regions. The highest coverage was observed in the south-western region, reaching 92.8% in 2003, while the lowest coverage was recorded in the northwest region in 2008, with a proportion of 20.6% (Table 4).

**Table 4:** Proportion of ANC coverage of at least four visits in Nigeria by Subnational region (2003, 2008, 2013 and 2018)

Country	Year	Subgroup	Estimate	SE	95%CI (Lower bound)	95%CI (Upper bound)
Nigeria	2018	north central	54.2	1.52	51.2	57.17
	2018	Northeast	43.98	1.65	40.78	47.23
	2018	North-west	42.25	1.49	39.35	45.2
	2018	South-east	82.92	1.11	80.64	84.99
	2018	South-south	69.46	1.58	66.27	72.48
	2018	South-west	84.24	1.14	81.88	86.35
	2013	north central	55.48	2.63	50.28	60.56
	2013	Northeast	38.85	2.23	34.58	43.31
	2013	North-west	30.35	1.66	27.2	33.7
	2013	South-east	82.91	1.69	79.34	85.96
	2013	south south	62.27	1.61	59.06	65.37
	2013	South-west	86.93	1.97	82.55	90.34
	2008	north central	48.28	2.15	44.08	52.5
	2008	Northeast	32.43	2.07	28.51	36.61
	2008	North-west	20.58	1.52	17.76	23.73
	2008	South-east	60.95	2.42	56.11	65.57
	2008	south south	53.26	1.95	49.42	57.06
	2008	South-west	80.69	1.73	77.06	83.87
	2003	north central	55.66	3.04	49.61	61.55
	2003	Northeast	32.53	2.46	27.87	37.55
	2003	North-west	28.56	2.15	24.51	32.99
	2003	South-east	72.16	6.55	57.67	83.14
	2003	south south	68.09	3.63	60.54	74.79
	2003	South-west	92.81	1.33	89.7	95.03

### ANC coverage at least four visits (%) by subnational region in Chad.

As shown in Table 5, the ANC coverage for at least four visits in Chad varied across different regions. The highest proportion was documented in N'djamena during the survey year 2014, reaching 57.8%, whereas the lowest proportion was observed in Biltine during the survey year 1997, with a mere 0.94% coverage (Table 5).

**Table 5:** Proportion of ANC coverage of at least four visits in Chad by Subnational region (1997, 2004 and 2014)

Country	Year	Subgroup	Estimate	SE	95%CI (Lower bound)	95%CI (Upper bound)
Chad	2014	Barh el gazal	20.17	4.66	12.52	30.84
	2014	Batha	12.56	3.46	7.19	21.05
	2014	Borkou/tibesti	23.55	6.74	12.87	39.12
	2014	Chari Baguirmi	12.75	2.49	8.61	18.49
	2014	Ennedi	14.32	2.8	9.65	20.73
	2014	Guera	31.59	6.12	20.94	44.6
	2014	Hadjer lamis	26.3	5.08	17.58	37.39
	2014	Kanem	22.89	3.67	16.49	30.87
	2014	Lac	13.06	3.75	7.28	22.32
	2014	Logone occidental	35.05	2.17	30.91	39.43
	2014	Logone oriental	38.86	4.39	30.66	47.75
	2014	Mandoul	29.06	3.64	22.46	36.68
	2014	Mayo kebbi est	38.38	2.86	32.94	44.13
	2014	Mayo kebbi oust	39.22	3.38	32.81	46.02
	2014	Moyen-chari	40.02	3.91	32.63	47.88
	2014	N'djamena	57.88	2.02	53.88	61.79
	2014	Ouaddai	14.57	3.67	8.73	23.33
	2014	Salamat	20.26	3.68	13.98	28.43
	2014	Sila	21.74	3.57	15.54	29.55
	2014	Tandjile	42.21	3.34	35.82	48.86
	2014	Wadi fira	12	2.01	8.57	16.55
	2004	Bar azoum	2.76	0.73	1.63	4.63
	2004	Borkou/tibesti	12.61	3.65	6.96	21.79
	2004	Centre est	9.77	2.82	5.42	16.97
	2004	Chari Baguirmi	9.74	2.45	5.84	15.8
	2004	Logone occidental	23.78	4.14	16.55	32.92
	2004	Mayo kebbi	19.07	2.47	14.65	24.44
	2004	Moyen-chari	15.11	3.78	9.02	24.22
	2004	N'djamena	57.75	2.32	53.09	62.27
	2004	Ouaddai est	4.64	1.36	2.57	8.22
	1997	Batha	1.83	0.93	0.67	4.93
	1997	Biltine	0.94	0.86	0.15	5.57
	1997	Borkou/tibesti	3.83	4.27	0.4	28.25
	1997	Chari Baguirmi	11.94	4.22	5.78	23.06
	1997	Guera	8.26	2.64	4.32	15.22
	1997	Kanem	12.63	5.15	5.43	26.69
	1997	Lac	3.69	1.7	1.47	8.98
	1997	Logone occidental	13.33	2.53	9.08	19.16
	1997	Logone oriental	19.74	2.77	14.82	25.8
	1997	Mayo kebbi	16.35	3.63	10.36	24.84
	1997	Moyen-chari	19.31	2.22	15.3	24.07
	1997	N'djamena	44.96	2.18	40.7	49.3
	1997	Ouaddai	4.28	1.03	2.65	6.85
	1997	Talamat	10.1	4.25	4.26	22.1
	1997	Tandjile	16.57	2.5	12.2	22.12

### ANC coverage at least four visits (%) by subnational region in Liberia and Sierra Leone

The proportion of ANC coverage of at least four visits in Liberia was reported to be high across regions in most survey years. The highest reported proportion is 89.9% in North Central in the survey year 2019 while the lowest was observed in 2007 in the southeastern a region. The proportion of ANC coverage in Sierra Leone was reported to be highest in the survey year 2019 with a proportion of 86.4% in the northwestern while the lowest was observed in the survey year 2008 in the north. (Table 6).

**Table 6:** Proportion of ANC coverage at least four visits in Liberia and Sierra Leone by Subnational region (2007, 2008, 2013 and 2019)

Country	Year	Subgroup	Estimate	SE	95%CI (Lower bound)	95%CI (Upper bound)
Liberia	2019	north central	89.99	1.47	86.72	92.53
	2019	Northwestern	87.62	2.46	81.92	91.71
	2019	south central	86.26	1.38	83.3	88.76
	2019	southeastern a	83.24	2.73	77.18	87.95
	2019	southeastern b	82.88	2.62	77.1	87.44
	2013	north central	79.19	1.95	75.1	82.76
	2013	Northwestern	76.78	2.17	72.24	80.78
	2013	south central	81.53	1.48	78.44	84.27
	2013	southeastern a	73.03	2.06	68.79	76.89
	2013	southeastern b	59.77	3.01	53.73	65.53
	2007	Monrovia	75.86	2.2	71.25	79.94
	2007	north central	63.41	3.26	56.77	69.57
	2007	Northwestern	67.48	3.6	60.01	74.17
	2007	south central	68.07	1.45	65.15	70.85
	2007	southeastern a	51.31	3.65	44.13	58.43
	2007	southeastern b	53.52	4.07	45.46	61.4
Sierra Leone	2019	East	84.59	1.44	81.55	87.21
	2019	North	85.57	1.84	81.57	88.82
	2019	Northwestern	86.41	1.44	83.34	89
	2019	South	78.26	1.69	74.76	81.39
	2019	West	59.39	3.23	52.93	65.54
	2013	East	73.22	3.2	66.48	79.04
	2013	North	74.53	1.47	71.53	77.32
	2013	South	80.45	1.73	76.82	83.63
	2013	West	77.65	2.98	71.25	82.96
	2008	East	60.78	2.71	55.33	65.96
	2008	North	49.35	2.21	45.02	53.68
	2008	South	56.64	2.42	51.84	61.32
	2008	West	69.35	2.55	64.11	74.13

### Inequality by age group

In Nigeria, from the survey conducted in 2003 revealed a significant level of inequality between age groups. Notably, it was observed that ANC coverage was higher among individuals aged 20–49 years (D = 14.8, PAF = 5.8, and PAR = 2.7) than those 15–19 years of age. Similarly, higher ANC coverage was observed in all the survey years 2008, 2013 and 2018 among age groups 20–49. In contrast, our analysis of Chad using summary measures such as PAF and PAR revealed no observed inequality across all survey years studied, namely 1997, 2004, and 2014, as both measures were found to be zero. However, the D measure, a value of -1, -2.4, and -3.4 was identified in the respective survey years. This indicates the presence of inequality, with higher ANC coverage observed among the age group 15–19.

In Liberia, no disparities were found in ANC coverage among different age groups during the survey years of 2007 and 2013, as indicated by the summary measures of PAF and PAR. However, when considering the D measure, values of -0.4 and -1.3 were observed, suggesting a slightly higher prevalence of ANC coverage in the age group 15–19. In contrast, during the survey year 2019, inequality was observed with a D value of 1.4, PAF value of 0.3, and PAR value of 0.3, indicating a slightly higher ANC coverage among individuals aged 20–49. In Sierra Leone, the survey conducted in 2008 revealed inequality in ANC coverage, with a D = 0.5, PAF = 0.1, and PAR = 0.1. This suggests a slightly higher ANC coverage among individuals aged 20–49. However, in the survey years of 2013 and 2019, no inequalities were observed between different age groups based on the PAF and PAR measures. Nevertheless, when considering the D measure, inequalities were observed with values of -2.2 in 2013 and -3.4 in 2019, indicating a noticeable difference in ANC coverage among various age groups during those years.

### Inequality by educational status

In Nigeria, inequality was observed in all the survey years with the four summary measures D, ACI, PAF and PAR, where the highest inequality was observed in the survey year 2003 with ACI = 12.7, D = 55.6, PAF = 70 and PAR = 33.2. This indicates the existence of inequality in ANC coverage where there exists higher coverage in the group with higher educational attainment (secondary school). Likewise, in Chad, significant disparities were observed in all the survey years using various measures such as ACI, D, PAF, and PAR. The highest level of inequality was identified in the 1997 survey, with ACI = 3.9, D = 45.8, PAF = 272.8, and PAR = 41. These values indicate the existence of inequality, where ANC coverage was notably higher among individuals with secondary educational attainment.

In Liberia, a higher level of inequality was observed in all the survey years with the four inequality measures. In 2007 and 2013 (ACI = 2.8, D = 15.6, PAF = 16.9, PAR = 11.1 and ACI = 3.6, D = 15.9, PAF = 10.7, PAR = 8.4) respectively indicating higher ANC coverage in the secondary school educational attainment subgroup. In contrast, in the survey year 2019, a slightly lower level of inequality was observed when compared with the previous survey years (ACI = 1.0, D = 4.1, PAF = 2.9 and PAR = 2.5). In Sierra Leone, it was found that there was a higher level of inequality of ANC coverage in the survey years 2008 and 2019 with (ACI = 2.2, D = 14.5, PAF = 21.0, PAR = 11.8 and ACI = 1.6, D = 8.3, PAF = 8.0, PAR = 6.1) respectively, indicating higher ANC coverage in the secondary school educational attainment subgroup. However, in the survey year 2019, higher inequality was observed in the lower educational attainment subgroup with ACI = -0.6, and D = -2.9, whereas there was no inequality with the measures PAF and PAR.

### Inequality by residence

In Nigeria, inequality was observed in all the survey years with D, PAF and PAR where in 2008 and 2013, (D = 34.4, PAF = 53.6, PAR = 24.0 and D = 36.6, PAF = 45.8, PAR = 23.4. These values indicate the existence of inequality, where ANC coverage was high among those who reside in the Urban area. In Chad, inequality was observed in all the survey years from 1997 to 2014 where the highest inequality was observed in the survey year 2004 (D = 31.9, PAF = 145.7 and PAR = 25.9). These values indicate the occurrence of inequality among people residing in the urban area, which means that those residing in the urban area has high ANC coverage than that of the rural one. In Liberia, inequality existed in all the survey years but was slightly lower than in Nigeria and Chad. As indicated with the summary measures D, PAF and PAR, in the consecutive survey years, 2007, 2013 and 2019 (D = 15.2, PAF = 15.4, D = 11.3, PAF = 6.7, PAR = 5.3 and D = 4.7, PAF = 2.4, PAR = 2.1) respectively.

In Sierra Leone, higher inequality in ANC coverage was observed in the survey years 2008 and 2013, with (D = 13.7, PAF = 17.3 and D = 5.3, PAF = 5.1, PAR = 3.8) respectively. This indicates that ANC coverage was higher in urban areas than in rural areas. In contrast, slightly lower to no inequality was observed in the survey year 2019, with a (D = -10.1, PAF = 0.0, PAR = 0.0). This suggests that ANC coverage was higher among those residing in rural areas. The summary measures PAF and PAR also indicate that inequality was non-existent with place of residence in 2019.

### Inequality by economic status

Inequality by economic status was measured by four summary measures, named ACI, D, PAF and PAR. It was observed that inequality has existed in all survey years in Nigeria where in 2003 (ACI = 12.7, D = 64.5, PAF = 85.7, PAR = 40.6), 2008 (ACI = 13.2, D = 64.9, PAF = 79.9, PAR = 35.8), 2013(ACI = 14, D = 67.6, PAF = 67.5, PAR = 34.5) and 2018(ACI = 11.1, D = 54.7, PAF = 50.2, PAR = 28.5). This shows the existence of inequality where there is high ANC coverage among people in the highest wealth quintile (richest).

In Chad, inequality was observed in each survey years where in 1997 (ACI = 6.2, D = 34.9, PAF = 164.3 and PAR = 24.,), 2004(ACI = , D = , PAF = and PAR = ,), and 2014(ACI = , D = , PAF = and PAR = ,). This implies that there is high inequality of ANC coverage by economic status where ANC coverage is found to be higher among those who were in the richest quintile Subgroup. In Liberia, inequality was observed by Economic status in all the survey years, where the highest was reported in the survey year 2007 (ACI = 5.2, D = 23.5, PAF = 18.8, and PAR = 12.4). The lowest was reported in the survey year 2019(ACI = 1.5, D = 8.8, PAF = 4.6, PAR = 4.0). All indicating the higher ANC coverage among the richest quintile subgroup.

In Sierra Leone, higher inequality was observed in the survey years 2008 and 2013 (ACI = 3.5, D = 21.2, PAF = 24.8, PAR = 13.9) and (ACI = 1.3, D = 6.3, PAF = 5.5, PAR = 4.2), which indicates higher inequality in ANC coverage where higher ANC coverage to be found among the richest quintile. Whereas in the survey year 2019 (ACI = -2.4, D = -11.4, PAF = 0.0, PAR = 0.0), indicating higher inequality of ANC coverage with ACI and D, where ANC coverage is found to be higher among the poorest quintile subgroup. On the other hand, PAF and PAR inequality measures showed no inequality among subgroups.

### Inequality by subnational region

In Nigeria, based on the summary measures of PAF and PAR in the survey year 2003(PAF = 95.7, PAR = 45.4), there is higher inequality in ANC coverage by the Subnational region. This indicates that there was higher ANC coverage in the southwestern region whereas the lowest coverage is in the north western region. In the survey years 2008, 2013 and 2018, the Southwestern region was mainly the one with the highest ANC coverage 2008(D = 60.1, PAF = 80.0, PAR = 34.9), 2013 (D = 56.6, PAF = 70.1, PAR = 35.8) and 2018 (D = 42.0, PAF = 48.2, PAR = 27.4); whereas the northwestern was the one with the lowest in all the survey years. In Chad, inequality was observed by subnational regions in all the survey years with (D = 44.0, PAF = 198.3, PAR = 29.9) in 1997 where biltine was the region with the lowest and ANC coverage while n'djamena was the one with the highest ANC coverage, (D = 55.0, PAF = 224.9, PAR = 40.0) in 2004 where ouaddai est was the one with the lowest while n'djamena was the one with the highest ANC coverage and (D = 45.9, PAF = 86.8, PAR = 26.9) in 2014 where wadi fira to be the region with the lowest coverage while n'djamena is the highest. In Liberia, higher inequality was observed in all the survey years by subnational region, 2007 (D = 24.6, PAF = 15.0, PAR = 9.9), 2013 (D = 21.8, PAF = 4.4, PAR = 3.4) and 2019(D = 7.1, PAF = 3.1, PAR = 2.7). The highest coverage is reported in Monrovia in 2007, south-central in 2013 and north-central in 2019. In Sierra Leonne, higher inequality by subnational region was observed in 2008,2013 and 2019 (D = 20.0, PAF = 23.6, PAR = 13.2), (D = 7.2, PAF = 5.8, PAR = 4.4) and (D = 27.0, PAF = 9.7, PAR = 7.7) respectively. The highest coverage was reported in the west in the survey year 2008, while the lowest was in the north. Whereas in the survey year 2013, the highest coverage was reported in the south, while the lowest was in the east. On the other hand, in the 2019 survey year, the highest coverage was observed in the northwestern, while the lowest was in the west.

### Intra-national inequality of ANC coverage

When we compare inequality in ANC coverage with Age, Nigeria showed the highest inequality with all the summary measures followed by Libera: D, PAF and PAR. Whereas Chad and Sierra Leonne exhibited slightly higher inequality with a high ANC coverage in the age group 15–19, with summary measure D and inequality was reported to be non-existent with PAF as well as PAR for these countries.

Nigeria also showed the highest inequality of ANC coverage by Economic status with summary measures ACI, D and PAR, while Chad exhibited the highest inequality with the summary measure PAF followed by Liberia. Sierra Leone showed higher ANC coverage in the poorest quintile status while the rest reported higher coverage in the richest wealth quintile. When we compare inequality by Education, Nigeria exhibited the highest inequality by the summary measures ACI and D where the ANC coverage was concentrated among educated, while Chad exhibited the highest inequality by summary measures PAF and PAR followed by Liberia. Whereas, Sierra Leonne reported a slightly higher ANC coverage among those with no formal education while the rest reported higher ANC coverage among those with Secondary school and above. Further, summary measures of PAF and PAR showed no inequality.

When we compare inequality with place of residence, Nigeria showed higher inequality favoring the urban residing community with summary measure D, while Chad exhibited higher inequality with the summary measures PAF and PAR followed by Nigeria and Liberia. Sierra Leonne further showed higher inequality with D among those residing in the rural areas and no inequality by residence with summary measures PAF and PAR. When we compare inequality by subnational region, Chad exhibited the highest inequality compared with Nigeria, Liberia and that of Sierra Leonne with summary measures D, PAF and PAR. Nigeria took the second place with the highest inequality while Sierra Leonne and Liberia took the third and fourth place, with all showing higher coverage (Table 7).

**Table 7:** Intra-nation ANC coverage with five Dimensions of four countries with the highest maternal mortality in Africa

Country	Year	Dimension	Summary metrics	Estimate	95% CI (Lower bound)	95% CI (Lower bound)
Nigeria	2018	Age (2 groups) (15–49)	D	11.6	8.3	14.9
Nigeria	2018	Age (2 groups) (15–49)	PAF	2.5	-0.6	5.6
Nigeria	2018	Age (2 groups) (15–49)	PAR	1.4	-0.4	3.2
Nigeria	2018	Economic status (wealth quintile)	ACI	11.1	10.5	11.7
Nigeria	2018	Economic status (wealth quintile)	D	54.7	51.6	57.9
Nigeria	2018	Economic status (wealth quintile)	PAF	50.2	48.1	52.3
Nigeria	2018	Economic status (wealth quintile)	PAR	28.5	27.3	29.8
Nigeria	2018	Education (3 groups)	ACI	10.9	10.3	11.5
Nigeria	2018	Education (3 groups)	D	44.6	42.2	46.9
Nigeria	2018	Education (3 groups)	PAF	39.1	37.8	40.5
Nigeria	2018	Education (3 groups)	PAR	22.2	21.5	22.9
Nigeria	2018	Place of residence	D	27.9	25.5	30.6
Nigeria	2018	Place of residence	PAF	29.6	28.6	30.6
Nigeria	2018	Place of residence	PAR	16.8	16.3	17.4
Nigeria	2018	Subnational region	D	42.0	38.3	45.7
Nigeria	2018	Subnational region	PAF	48.2	46.6	49.8
Nigeria	2018	Subnational region	PAR	27.4	26.5	28.3
Chad	2014	Age (2 groups) (15–49)	D	-3.4	-7.1	0.2
Chad	2014	Age (2 groups) (15–49)	PAF	0	-5.9	5.9
Chad	2014	Age (2 groups) (15–49)	PAR	0	-1.8	1.8
Chad	2014	Economic status (wealth quintile)	ACI	4.6	3.7	5.5
Chad	2014	Economic status (wealth quintile)	D	29.8	24.6	34.9
Chad	2014	Economic status (wealth quintile)	PAF	72.7	67.6	77.9
Chad	2014	Economic status (wealth quintile)	PAR	22.5	20.9	24.1
Chad	2014	Education (3 groups)	ACI	5.8	5.2	6.6
Chad	2014	Education (3 groups)	D	34.9	30.5	39.2
Chad	2014	Education (3 groups)	PAF	85.8	83.6	87.9
Chad	2014	Education (3 groups)	PAR	26.6	25.9	27.2
Chad	2014	Place of residence	D	25.1	20.9	29.3
Chad	2014	Place of residence	PAF	64.8	63.2	66.4
Chad	2014	Place of residence	PAR	20.1	19.6	20.6
Chad	2014	Subnational region	D	45.9	40.3	51.5
Chad	2014	Subnational region	PAF	86.8	74.0	99.6
Chad	2014	Subnational region	PAR	26.9	22.9	30.9
Liberia	2019	Age (2 groups) (15–49)	D	1.4	-2.4	5.2
Liberia	2019	Age (2 groups) (15–49)	PAF	0.3	-2.1	2.8
Liberia	2019	Age (2 groups) (15–49)	PAR	0.3	-1.8	2.4
Liberia	2019	Economic status (wealth quintile)	ACI	1.5	0.7	2.3
Liberia	2019	Economic status (wealth quintile)	D	8.8	4.1	13.5
Liberia	2019	Economic status (wealth quintile)	PAF	4.6	2.1	7.1
Liberia	2019	Economic status (wealth quintile)	PAR	3.9	1.8	6.2
Liberia	2019	Education (3 groups)	ACI	1.1	0.3	1.8
Liberia	2019	Education (3 groups)	D	4.1	0.8	7.4
Liberia	2019	Education (3 groups)	PAF	2.9	1.2	4.6
Liberia	2019	Education (3 groups)	PAR	2.5	1.1	3.9
Liberia	2019	Place of residence	D	4.7	1.4	8.1
Liberia	2019	Place of residence	PAF	2.4	0.9	3.7
Liberia	2019	Place of residence	PAR	2.1	0.9	3.3
Liberia	2019	Subnational region	D	7.1	1.2	13.0
Liberia	2019	Subnational region	PAF	3.1	-2.4	8.6
Liberia	2019	Subnational region	PAR	2.7	-2.1	7.5
Sierra Leone	2019	Age (2 groups) (15–49)	D	-3.4	-6.6	-0.1
Sierra Leone	2019	Age (2 groups) (15–49)	PAF	0	-2.6	2.6
Sierra Leone	2019	Age (2 groups) (15–49)	PAR	0	-2.1	2.1
Sierra Leone	2019	Economic status (wealth quintile)	ACI	-2.4	-3.5	-1.4
Sierra Leone	2019	Economic status (wealth quintile)	D	-11.8	-18.1	-5.6
Sierra Leone	2019	Economic status (wealth quintile)	PAF	0	-2.2	2.2
Sierra Leone	2019	Economic status (wealth quintile)	PAR	0	-1.8	1.8
Sierra Leone	2019	Education (3 groups)	ACI	-0.6	-1.6	0.3
Sierra Leone	2019	Education (3 groups)	D	-2.8	-6.9	1.1
Sierra Leone	2019	Education (3 groups)	PAF	0	-1.1	1.1
Sierra Leone	2019	Education (3 groups)	PAR	0	-0.9	0.9
Sierra Leone	2019	Place of residence	D	-10.1	-14.4	-5.8
Sierra Leone	2019	Place of residence	PAF	0	-0.9	0.9
Sierra Leone	2019	Place of residence	PAR	0	-0.8	0.8
Sierra Leone	2019	Subnational region	D	27.0	20.1	33.9
Sierra Leone	2019	Subnational region	PAF	9.7	6.9	12.5
Sierra Leone	2019	Subnational region	PAR	7.7	5.5	9.9

## Discussion

This study assessed the demographic and economic inequities in antenatal care coverage based in four high maternal mortality countries in African. [38]. Key social determinants of inequalities in ANC coverage, including women’s education, age group, residency, and wealth explain the significant socioeconomic inequalities in ANC care coverage in these four African countries.

A survey conducted in four African countries showed that higher ANC coverage was observed in all the survey years among age groups 20–49 years than those with age group of 15–19 age group which is higher in Nigeria and Sierra Leone. The possible reasons could be older mothers may have a higher level of awareness regarding the importance of antenatal care and higher degree of consciousness regarding their health and well-being due to their life experience and exposure to healthcare information [39, 40]. On the other side study in Chad and Liberia revealed the presence of inequality, with higher ANC coverage observed among the age group 15–19, this might be due to adolescents may have higher health risks and complications during pregnancy compared to older women. This may lead to additional health concerns and a greater need for antenatal care [41].

The existence of higher ANC coverage in the group with higher educational attainment (secondary school) in Nigeria, Chad, Liberia, and Sierra Leone. An educated mother is more use the higher level of ANC utilization compared to a mother with no education, being consistent with previous studies [42]. These findings highlight the role of education system in promoting more ANC coverage [43, 44]. Also, formal education tends to improve and promote women’s decision-making and overall empowerment, thereby making them more confident to demand adequate ANC services [40, 45].

There is an existence of ANC coverage was high among those who reside in the urban area, in contrast, in the survey year 2019 suggests that ANC coverage was higher among those residing in rural areas. This urban–rural inequality in maternal health care service uptake is a major concern. This finding is common in many resource-constrained settings due to disparity in the distribution of functional health facilities which is usually in favor of the urban residence [46, 47]. In the rural area, poor medical service content was the main reason for the low proportion of overall adequate ANC. The main reason for low ANC adequacy among rural women was insufficient use of core ANC services. This might happen because health providers are not able to offer appropriate service [44]. Also rural set-up face barriers of transportation and reaching the health facility to receive appropriate antenatal care [4]. This warrants the need for attention to urban–rural.

There was high ANC coverage among people in the highest quintile status (richest), in Chad, in Liberia, and in Sierra Leone. This implies that wealth status had a significant impact on uptake of all three types of MHS in the study population. Compared with the women in the poorest wealth quintile, those in the higher quintile have significantly higher odds of receiving at least four ANC visits, our results are consistent with previous findings [4, 45]. Women from wealthy households are able to afford health care services especially in out-of-pocket health expense situation [44, 47, 48].

There was also inequality among subnational region at the different survey years in Nigeria, Chad, Liberia and Sierra Leone. In Nigeria, higher ANC coverage was observed in the southwestern region whereas the lowest coverage is in the north western region. The likely explanation for this disparity could be that the northern region has a lower proportion of women with formal education, leading to an increase in the number of women who lack knowledge about the importance of ANC services [49]. While in Chad, N’Djaména exhibited higher ANC coverage than that of the other regions in the different survey years. The main reason behind this difference could be attributed to the urban nature of N'Djaména. As Chad's capital and largest city, N'Djaména possibly offers a higher likelihood of accessibility to the service due to the presence of a concentrated pool of professionals and facilities that cater to the needs of the population. The finding from this study has also shown that Monrovia, South-central and North-central of Liberia exhibited higher ANC coverage than the others. The possible explanation for this could be the betterment in the economic(quintile) status of the regions compared to the others in the survey years [50].

When we compare inequality in ANC coverage with Age, Nigeria showed the highest inequality followed by Liberia. As we see the age group difference in Nigeria is higher than that of Liberia so that it causes one factor for the ANC care coverage. This might be also difference in cultural because in some cultures, older women are accorded more respect and may be given higher priority when it comes to accessing healthcare services [51]. One of the significant factors that can affect the uptake of antenatal care (ANC) is cultural differences among countries. It is essential to acknowledge that cultural differences can significantly impact the utilization of antenatal care services. Cultural difference included cultural beliefs and attitudes, gender roles, barriers in accessing ANC, and prioritizes traditional healers over formal healthcare facilities [52]. The impact of cultural factors on a woman's beliefs regarding antenatal care (ANC) and pregnancy, as well as her capacity to make autonomous healthcare choices, has been demonstrated to be significant, for example the belief that women did not need to book early for ANC since they do not have any problems in early pregnancy that need a doctor’s intervention or the presumption that there is no benefit in booking in the first three months was found to affect the uptake [53]. Compared to Liberia, Nigeria is a multicultural and multi-ethnic society [54] that needs an understanding of the cultural context by healthcare providers to provide ANC services in local languages and ensure cultural competence. Some women held the belief that revealing their pregnancy prematurely could put their unborn child at risk or allow enemies to bewitch them, resulting in a miscarriage. This belief led to the late initiation of antenatal care [55]. In certain cultures, it is customary for a woman's mother-in-law to determine whether or not she is eligible to receive care [56, 57]. Hence, it is crucial to consider these differences when developing strategies to improve ANC uptake.

## Conclusion and recommendation

The findings of the study revealed significant socioeconomic inequalities in antenatal care (ANC) coverage in four high-mortality African countries: Nigeria, Chad, Liberia, and Sierra Leone. The study demonstrates that women from socioeconomically disadvantaged households, lower educational attainment, and age of mother were factors associated with these inequalities. Addressing the socioeconomic inequalities in ANC coverage in Nigeria, Chad, Liberia, and Sierra Leone are crucial for improving maternal health outcomes and achieving the Sustainable Development Goals. Targeted interventions that focus on improving access to ANC services for women from disadvantaged backgrounds, promoting education and awareness, strengthening healthcare systems, and addressing social determinants of health are essential to reduce the disparities and ensure equitable maternal healthcare for all women in these countries.

## Strength and limitation

The study highlights the importance of addressing the disparities of ANC coverage respected with demographic and economic inequalities to improve maternal health outcomes and achieve the Sustainable Development Goals, however, the study relies on survey data collected over multiple years. Hence, the accuracy and reliability of the findings may be influenced by the quality of the data sources and potential variations in data collection methods. Further, the inconsistency in the number of data points for each country which is six data points for Nigeria, while three for Liberia, Chad and Sierra Leone due to the survey year variation is the limitation of the current study.

## Data Availability

The datasets generated and/or analysed during the current study are available in the WHO HEAT version 3.1 softwarerepository (https://whoequity.shinyapps.io/HEAT/).
